# Five-Year Overall Survival of Interval Breast Cancers is Better than Non- Interval Cancers from Korean Breast Cancer Registry

**DOI:** 10.31557/APJCP.2019.20.6.1717

**Published:** 2019

**Authors:** Jung Sun Lee, Hyun-Ah Kim, Se-Heon Cho, Han-Byoel Lee, Min Ho Park, Joon Jeong, Heung Kyu Park, Minkyung Oh, Onvox Yi

**Affiliations:** 1 *Department of Surgery, Haeundae Paik Hospital, College of Medicine, Inje University, *; 3 *Department of Surgery, College of Medicine, Dong- A University,*; 8 *Department of Pharmacology, Inje University College of medicine, Clinical Trial Center, Inje University Busan Paik Hospital, *; 9 *Department of Surgery, Dongnam Institution of Radiological and Medical Science, Busan, *; 2 *Department of Surgery, Korea Cancer Center Hospital, Korea Institute of Radiological and Medical Sciences, *; 4 *Department of Surgery, Seoul National University College of Medicine, *; 6 *Department of Surgery, Gangnam Severance Hospital, Yonsei University Medical College, Seoul, *; 5 *Department of Surgery, Chonnam National University Hwasun Hospital, Chonnam, *; 7 *Department of Surgery, Breast Cancer Center, Gachon University Gill Medical Center, Incheon, Korea. *

**Keywords:** Breast neoplasm, early detection of cancer, mammography, health care disparities, prognosis

## Abstract

**Objective:**

Interval breast cancer (IC) is a limitation of breast cancer screening. We investigated data from a large scaled breast cancer dataset of patients with breast cancer who underwent breast cancer screening in order to recapitulate the overall survival (OS) of patients with ICs compared to those with non-ICs.

**Methods:**

A total of 27,141 patients in the Korean breast cancer registry with breast cancer who had ever participated in biannual national breast cancer screening programs between 2009 and 2013 were enrolled. We compared the social, pregnancy-associated, and pathologic characteristics between the IC and non-IC groups and identified the significant prognostic factors for OS.

**Results:**

The proportion of ICs was 1.3% (370/27,141) in this study population. ICs were correlated with age 45-55 years at diagnosis, higher levels of education, early menopause (<50 years), hormone replacement therapy, specific provinces (Kangwon, Kyungnam, Jeju, and Dae-jeon), and family history of breast cancer. Low-to-intermediate nuclear grade, early stage (stage 0-I), and low Ki-67 level were also correlated with IC proportion. Non-ICs were associated with an increased risk of five-year mortality (hazard ratio [HR] 7.4; 95% confidence interval [CI]:1.85-29.66; p = 0.005) compared to ICs. Lymph node metastasis, residence (Kyung-nam province), low education status, high histologic grade, and asymptomatic cancers increased the HR of five-year OS.

**Conclusion:**

ICs occurred unequally in specific province and relatively high-educated women in Korea. They were also diagnosed with early-stage breast cancer with a favorable recurrence risk, and their outcome was better than those of patients with other breast cancers in breast cancer screening.

## Introduction

When a mammography screening program is fully implemented, interval cancers comprise a substantial proportion of incident breast cancers. Interval cancers could have been overlooked at the previous mammography examination or become apparent because they grew rapidly such that the detectable preclinical phase was shorter than that of the screening interval. In Korea, the incidence rate of interval breast cancers (ICs) per 100,000 negative findings increased from 51.7 in 2009 to 76.3 in 2014 (Lee et al., 2016). Although research on interval cancer of mammography screening has focused on survival comparing to different groups. Several studies (Collett et al.,2005; Bellio et al., 2005; Sihto et al., 2008) have reported that the prognosis of ICs considered is poorer than that of screening-detected breast cancers because IC tumors are, on average, larger than those of more advanced cancers, and tried to identify women at risk of ICs. Previous randomized trials on mammography screening found that ICs were associated with similar (Holmberg et al., 1986; Delacour-Billon et al., 2017), better (Frisell et al., 1992) or poorer (Andersson et al., 1998; Musolino et al., 2018) survival compared to that of non-screened breast cancers. Kalager et al., (2012) reported in an observational cohort study that the prognosis of women with ICs was the same as that of women with breast cancer diagnosed without mammography screening. They concluded that IC tumors are more likely to be larger than those of non-screening-detected cancers, but have similar survival outcomes and provided no compelling support for more aggressive primary treatment of ICs than non-screening-detected cancers.

The National Health Insurance Service (NHIS) in Korea began screening for breast cancer using mammography once every two years without copayment in 1999, targeting women with medical aid who were over 40 years of age. An increasing tendency in screening rate in Korea was observed before 2012 (70.2%), which decreased from 2012 to 2014 (59.3%) in all age groups (Suh et al., 2016). These decreasing rates may be due to negative press messages about mammography screening in Korea since 2012. In addition, the limitations of screening mammography include a painful procedure, high false-positive rates, and misdiagnosis, especially for ICs in younger women (Lee et al., 2016; Kim and Kim, 2016; Suh et al., 2016). Therefore, this study evaluated patients who have ever breast cancer screening before a diagnosis of breast cancer for determining the frequency of reported ICs, associated reproductive or social factors, short term-clinical outcomes and for highlighting the risk of ICs from a cancer registry database.

## Materials and Methods

*Study cohort*

**Figure 1:**
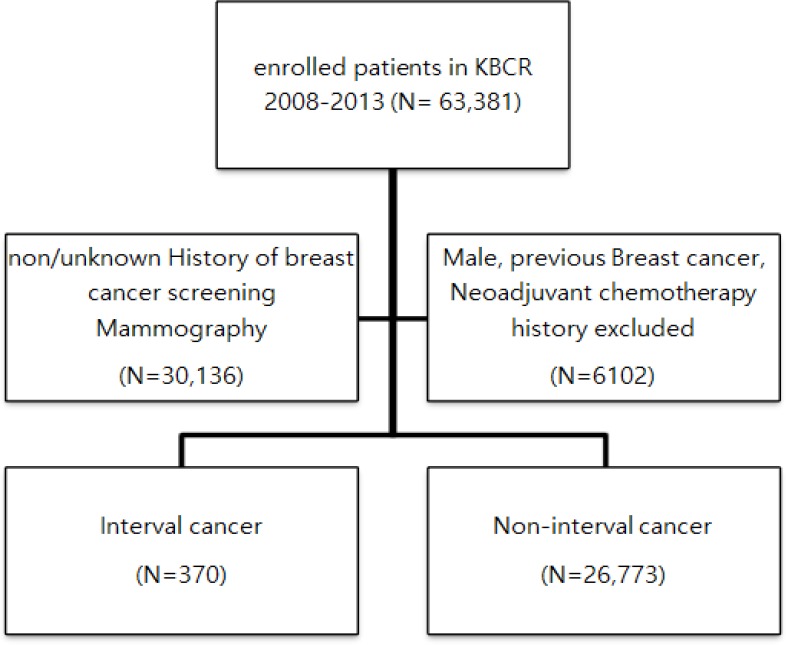
Flow Chart Representing the Selection Procedure Based on the KBCR Dataset from 2009-2013

The KBCR is a prospectively maintained, web-based database of the Korean Breast Cancer Society (Moon et al., 2009; Moon et al., 2010). The registry is estimated to include more than 65% of all newly diagnosed breast cancer patients in Korea in 2013 (Min et al., 2016). Between January 2009 and December 2013, a total of 63,381 patients were enrolled by 102 general hospitals, including 41 university hospitals and 61 surgical training hospitals. Patients with male sex, previous history of breast cancer, or treatment with neoadjuvant chemotherapy were excluded. The history of screening by mammography was unknown in almost half of these cases; after excluding these cases, a total of 27,143 (42.8%) patients were included in the final analysis. Cancer that occurs within 12 months after negative results on cancer screening are defined interval cancer and we classified 27,143 patients by status of interval breast cancer (Figure 1). Personal interviews were conducted with each patient at the time of diagnosis to generate information about each subject, including demographic information, reproductive variables (age at menarche, menopause, pregnancy, childbirth, and age at first birth [AFB]). The database provided information about sex, age, type of operation, stage according to 6th American Joint Committee on Cancer classification, histological findings and the presence of biological markers, adjuvant therapy, and status of interval cancer was reported by physicians. Status of survival and cause of death until 31, Dec, 2014, which was obtained from the Ministry of Health and Welfare, Republic of Korea. The mean duration of follow-up was 40.17 months (±17.48, 11.9-72.73). This study was approved by the Institutional Review Board (IRB number: HPIRB 2017-07-639-001) and was conducted in accordance with the Declaration of Helsinki.

*Statistical analysis*

The clinicopathologic characteristics of ICs and non-ICs were compared using Х^2^ and t-tests. Multivariate Cox proportional hazard models were used to determine the effect of interval breast cancer on disease-free survival (DFS) (or death due to breast cancer) and overall survival (OS) (deaths from any cause) rates as dependent variables and adjusting for age and stage. Hazard ratios (HR) and 95% confidence intervals (CI)s were calculated for the following study factors: age at diagnosis (<35, 35-44, 45-54, ≥55); menarche (<13, 13-15, ≥16); tumor size (≤1cm, 1.1-2.0cm, ≥2.1cm); number of child (0-1, 2-3, ≥4); educational level ( high school, elementary, college, none, middle school); family history ( no, yes), and hormone replacement therapy( never, ever). We identify all statistical methods and verified the assumptions for all statistical tests which are performed by two-sided. All analyses were performed using SAS (Version 9.4, SAS, Inc., Cary, NC). Alpha for all statistical tests was 0.05. Statistical significance was assumed for p < 0.05.

## Results

*Characteristics of IC in KBCR data*

**Figure 2:**
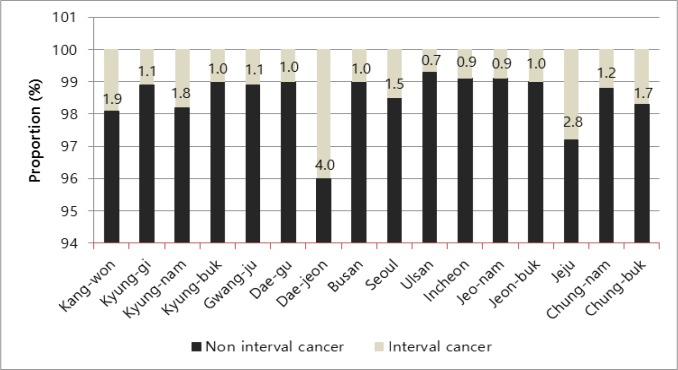
Regional Distribution of Interval Breast Cancer in Korea (2009-2013)

The proportion of ICs was 1.36 % (370/27,143). The proportion of ICs was correlated with age between 45 and 55 years at diagnosis, between 25 and 34 years at first birth, and family history of breast cancer. In menopausal patients with breast cancers, early menopause (<50 years) and use of hormonal replacement therapy were correlated with the proportion of ICs. The proportion of ICs was also significantly higher in patients with higher levels of education (Table 1) and in specific provinces (Kyung-nam, Dae-jeon, Jeju, Kang-won) (Figure 2). Marriage status, breastfeeding, use of oral contraceptives, age at menarche, parity, symptomatic disease, and body mass index (BMI) were not correlated with ICs. Regarding tumor characteristics, ICs were also correlated with low-to-intermediate nuclear grade, early stage (stage 0-I), negative Her-2/neu expression, and low Ki-67 levels. However, tumor size did not differ significantly between the two groups. The operation methods also differed, with breast-conserving surgery and sentinel lymph node biopsy highly correlated with ICs (Table 2). 

*DFS and associated factors in patients with breast cancer with mammography screening*

Patients with breast cancer who had never received adjuvant endocrine therapy or asymptomatic women showed highest HR of five-year DFS. Conversely, age at diagnosis (45-55) had a decreased HR of five-year DFS (HR 0.13; 95% CI:0.05-0.35; p <0.001) compared those of the other age groups. Women who entered menarche at 13-15 years of age showed a decreased HR of five-year DFS (HR 0.16; 95%CI: 0.06-0.42; p = 0.0002) compared to that in the other age groups. Compared to high parity (≥4), parity (≤3) had a decreased HR of five-year DFS. The educational level also affected the prognosis. A high educational level (above high school) was associated with a decreased HR of the five-years DFS (HR 0.13; 95%CI:0.02-0.69; p = 0.01) compared to that of other educational levels. Negative lymph node metastasis showed a relatively good prognosis compared to that of positive lymph node metastasis (HR 0.12; 95% CI:0.05-0.30; p <0.001). The other clinical factors and residence did not show prognostic effects on the five-year DFS (Table 3). Because of the short follow-up period and low frequency of deaths, we could not perform a stratified analysis between the IC and non-IC groups.

*OS and associated factors in patients with breast cancer with mammography screening*

**Figure 3:**
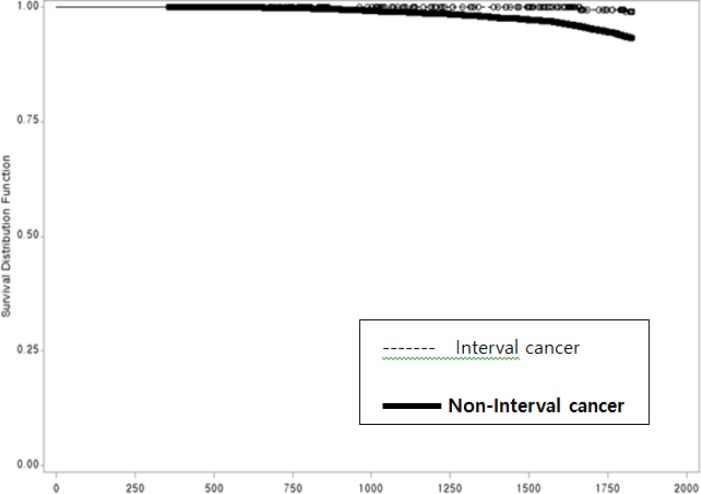
Kaplan- Meier Curve of 5 Years Overall- Survival

Compared to ICs, non-ICs increased the HR of a five-year OS (HR 7.4; 95%CI: 1.85-29.66; p = 0.005)(Figure 3). Residence (Kyung-nam province), low education status (non-educated or elementary school), high histologic grade, asymptomatic cancers, and patients without adjuvant endocrine therapy had an increased HR of five-year OS. Age at diagnosis (35-44 years) or (45-54 years) decreased the HR of five-year OS (HR 0.55; 95%CI: 0.44-0.68; p <0.001), (HR 0.52; 95%CI: 0.43-0.63; p <0.001). Women who entered menarche at 13-15 years of age decreased HR of five-year OS (HR 0.70; 95%CI: 0.58-0.83; p <0.0001). Compared with high parity (≥4), any parity (≤ 3) including nulliparous women, decreased HR of five-year OS. Negative lymph node metastasis showed a relatively good prognosis compared to that of positive lymph node metastasis (HR 0.29; 95%CI: 0.24-0.34; p <0.001). Other known prognostic factors (Ki-67, lymphovascular invasion, Her-2/neu, and tumor size) showed a negative correlation with the five-year OS (Table 4).

## Discussion

The cancer screening rate in this study (42.8%) was lower than that reported in a National Breast Cancer Screening Project report (59.3%) (Suh et al., 2016). Even though of enrollment younger than 40 years women in the cancer registry, it could be a considerable low screening rate. This finding reflects trends in decreasing screening rates. Korean women may prefer to check their breast by ultrasonography because personal invitation letter after the screening was inconclusive, disbelief of the findings, or discomfort regarding screening mammography (Kim et al., 2017). 

We observed a very lower proportion of ICs (1.3%) from registry data in the same period. About the incidence of ICs in Korea, it has been reported that it increased from 5.2 persons in 2009 to 7.8 persons per 10,000 negative mammography findings in 2014 (Lee et al., 2016), similar to those reported in other countries (Kemp Jacobsen et al., 2015). But direct comparison with this study was not appropriate because this study was from a large-scale observational study of Korean NHISS, it did not use actual biannual screening interval, but current study was from registry data, used actual screening interval (2 years). Actual incidence rate of ICs need to re-analyze with an actual screening interval from Korean breast cancer screening program. We started it through big data sharing program from Korean NHISS. 

**Table 1:** Comparison of Social Characteristics between Non-IBC and IBC among Patients with Breast Cancers Who Attended Ever Breast Cancer Screening

		Non IC (Case/ %)	IC (Case/ %)	Total	p-value^a^
Age at diagnosis	<35	1,181 (4.45)	9 (2.51)	1,190 (4.3)	<0.006
	35≤ <45	6,646 (25.05)	85 (23.74)	6,731 (24.8)	
	45≤ <55	10,545 (39.74)	178 (49.72)	10,723 (39.5)	
	≥55	8,164 (30.77)	86 (24.02)	8,250 (31.4)	
BMI( Kg/m^2^)	<18.5	1,534 (5.78)	25 (6.98)	1,559 (5.7)	0.735
	18.5-24.9	17,462 (65.8)	235 (65.64)	17,697 (64.5)	
	25-29.9	6,403 (24.13)	83 (23.18)	6,486 (23.9)	
	30-34.9	1,005 (3.79)	12 (3.35)	1,017 (3.7)	
	≥35	132 (0.5)	3 (0.84)	135 (0.5)	
Menarche	<13	5,791 (21.82)	68 (18.99)	5,859 (21.6)	0.106
	13-15	14,001 (52.76)	209 (58.38)	14,210 (52.3)	
	≥16	6,744 (25.41)	81 (22.63)	6,825 (25.1)	
Menopause	<50	20,677 (77.92)	288 (80.45)	20,965 (77.2)	0.006
	50-54	4,847 (18.27)	68 (18.09)	4,915 (17.9)	
	≥55	1,012 (3.81)	2 (0.56)	1,014 (3.7)	
Number of child	0-1	8,159 (61.78)	85 (63.91)	8,244 (61.8)	0.07
	2-3	3,546 (26.85)	41 (30.83)	3,587 (26.9)	
	≥4	1,502 (11.37)	7 (5.26)	1,509 (11.3)	
Age at first birth	≤24	12,040 (45.37)	140 (39.11)	12,180 (45.3)	0.024
	25-34	13,754 (51.83)	211 (58.94)	13,965 (51.9)	
	≥35	742 (2.8)	7 (1.96)	749 (2.8)	
Education level	High school	9,077 (39.78)	117 (36.79)	9,194 (39.7)	0.02
	Elementary	2,607 (11.43)	26 (8.18)	2,633 (11.4)	
	College	7,778 (34.09)	133 (41.82)	7,911 (34.2)	
	None	507 (2.22)	3 (0.94)	510 (2.2)	
	Middle school	2,847 (12.48)	39 (12.26)	2,886 (12.5)	
Marriage status	No	1,864 (7.21)	19 (5.38)	1,883 (7.2)	0.18
	Yes	23,978 (92.79)	334 (94.62)	24,312 (92.8)	
Breast feeding	No	7,408 (31.44)	96 (28.15)	7,504 (31.4)	0.19
	Yes	16,154 (68.56)	245 (71.85)	16,399 (68.6)	
Oral Contraceptive	No	21,449 (88.7)	290 (86.8)	21,739 (88.5)	0.27
	Yes	2,725 (11.3)	44 (13.2)	2,769 (21.5)	
HRT	No	22,619 (91.04)	287 (85.42)	22,906 (90.9)	0.0004
	Yes	2,227 (8.96)	49 (14.58)	2,276 (9.1)	
Family history	No	23,688 (90.56)	300 (87.21)	23,988 (90.5)	0.03
	Yes	2,469 (9.44)	44 (12.79)	2,513 (9.5)	
Symptom	No	18,597 (70.08)	203 (56.7)	18,800 (69.9)	<0.001
	Yes	7,939 (29.92)	155 (43.3)	8,094 (30.1)	

BMI, Body Mass Index; HRT; Hormonal Replacement Therapy; IC, Interval Cancer; ^a^p values were calculated using the Х^2^ and t-tests.

**Table 2:** Comparison of Tumor Characteristics between Non-IBC and IBC

		Non IC (Case/ %)	IC (Case/ %)	Total	p-value^b^
Tumor size	≤1cm	7695 (29)	108 (30.17)	7,803 (29.0)	0.24
	1-2cm	9,352 (35.24)	137 (38.27)	9,489 (35.3)	
	>2cm	9,489 (35.76)	113 (34.56)	9,602 (35.7)	
Stage	0	3,319 (12.51)	53 (14.8)	3,372 (13.0))	<0.001
	I	11,069 (41.43)	174 (47.03)	1,1243 (43.4)	
	II	8,558 (31.9)	98 (26.5)	8,656 (33.4)	
	III	2,331 (8.7)	26 (7.0)	2,357 (9.1)	
	IV	281 (1.06)	2 (0.5)	283 (1.1)	
Operation_breast	BCS	16,880 (64.02)	255 (71.23)	17,135 (64.1)	0.03
	MRM	9,342 (35.43)	101 (28.21)	9,443 (35.3)	
Operation_axilla	ALND	4,108 (15.51)	43 (12.01)	4,151 (15.5)	0.012
	SLNB	1,578 (57.31)	236 (65.92)	15,414 (57.4)	
	SLNB+ALND	5,066 (19.13)	54 (15.08)	5,120 (19.1)	
	None	2,130 (8.04)	25 (6.98)	2,155 (8.0)	
ER	Negative	7,246 (28.53)	96 (27.35)	7,342 (28.7)	0.22
	Positive	17,959 (70.72)	255 (72.65)	18,214 (71.3)	
PR	Negative	9,958 (39.27)	148 (42.17)	10,106 (39.6)	0.16
	Positive	15,209 (59.98)	203 (57.83)	15,412 (60.4)	
HER2overexpession	Negative	18,443 (69.5)	272 (75.98)	18,715 (80.1)	<0.001
	Positive	5,566 (20.98)	78 (21.79)	5,644 (19.9)	
Ki-67	low	2,695 (13.53)	41 (23.43)	2,736 (13.6)	<0.001
	high	17,228 (86.47)	134 (76.57)	17,362 (86.4)	
Chemotherapy	No	9,919 (40.19)	165 (47.41)	10,084 (40.3)	0.006
	Yes	14,759 (59.81)	183 (52.59)	14,942 (59.7)	
Radiotherapy	No	7,117 (29.61)	75 (21.37)	7,192 (29.5)	0.001
	Yes	16,915 (70.39)	276 (78.63)	17,191 (70.5)	
Endocrine therapy	No	7,339 (31.68)	96 (27.59)	7,435 (31.7)	0.1
	Yes	15,829 (68.32)	252 (72.41)	16,081 (68.3)	

ER, Estrogen Receptor; PR, Progesterone Receptor, HER-2; Human Epidermal Receptor-2/ neu; IC, Interval Cancer; ^b^p values were calculated using the Х^2^ and t-tests.

**Table 3:** HRs with Corresponding 95% CI s of Tumor Characteristics in 5-Years Disease Free Survival

		HR	95% CI		p-value^c^
Age at diagnosis	<35				
	35≤ <45				
	45≤ <55	0.136	0.05	0.35	<0.001
	≥55	1	ref		
Menarche	<13	0.585	0.24	1.4	0.22
	13-15	0.16	0.06	0.42	0.0002
	≥16	1	ref		
Tumor size	≤1cm	0.96	0.44	2.09	0.92
	1.1-2.0 cm	0.35	0.13	0.98	0.04
	≥2.1 cm	1	ref		
Number of child	0-1	0.15	0.062	0.409	0.0001
	2-3	0.2	0.072	0.599	0.003
	≥4	1	ref		
Education level	High school	0.13	0.02	0.69	0.01
	Elementary	2.35	0.81	6.77	0.11
	College	0.18	0.03	0.93	0.04
	None	0.94	0.11	8.06	0.95
	Middle school	1	ref		
HRT	No	1.22	0.28	5.19	0.78
	Yes	1	ref		
Family history	No	2.49	0.34	18.34	0.36
	Yes	1	ref		
Symptom	No	3..21	1.12	9.19	0.03
	Yes	1	ref		
LN metastasis	No	0.12	0.05	0.3	<0.001
	Yes	1	ref		
Endocrine therapy	No	6.55	2.4	17.88	0.0002
	Yes	1	ref		

HRT, Hormonal Replacement Therapy; HR, Hazard Ratio; CI, Confidence Interval; LN, Lymph Node; ^c^p values were calculated using the Х^2^ and t-tests.

**Table 4:** HRs with Corresponding 95% CI s of Tumor in 5-Years Overall Survival

		HR	95% CI		p-value^d^
Age at diagnosis	<35	0.78	0.55	1.12	0.19
	35≤ <45	0.55	0.44	0.68	<0.001
	45≤ <55	0.52	0.43	0.63	<0.001
	≥55	1	ref		
Menarche	<13	0.99	0.8	1.23	0.96
	13-15	0.7	0.58	0.83	0.0001
	≥16	1	ref		
Tumor size	≤1cm	0.36	0.29	0.045	<0.0001
	1.1-2.0cm	0.32	0.26	0.39	<0.0001
	≥2.1cm	1	ref		
Number of child	0-1	0.7	0.53	0.92	0.012
	2-3	0.56	0.41	0.77	0.0004
	≥4	1	ref		
Education level	High school	0.95	0.72	1.25	0.74
	Elementary	1.58	1.17	2.15	0.003
	College	0.77	0.58	1.04	0.09
	None	2.2	1.43	3.38	0.0003
	Middle school	1	ref		
HRT	No	1.17	0.87	1.57	0.28
	Yes	1	ref		
Family history	No	1.03	0.78	1.38	0.79
	Yes	1	ref		
Symptom	No	2.69	2.16	3.35	<0.0001
	Yes	1	ref		
Province	Kangwon	1.47	0.7	3.05	0.3
	Kyung-gi	1.24	0.7	2.2	0.44
	Kyung-nam	1.94	0.02	3.69	0.04
	Kyung-buk	1.18	0.58	2.41	0.64
	Gwang-ju	0.68	0.28	1.65	0.4
	Dae-gu	1.82	0.92	3.59	0.08
	Dae-jeon	0.92	0.43	1.93	0.83
	Busan	1	0.52	1.93	0.97
	Seoul	1.43	0.81	2.52	0.21
	Ulsan	1.76	0.93	3.35	0.08
	Incheon	1.65	0.88	3.08	0.11
	Jeo-nam	1.3	0.63	2.65	0.46
	Jeon-buk	1.74	0.94	3.23	0.07
	Jeju	1.11	0.31	3.91	0.86
	Chung-nam	1.31	0.67	2.56	0.42
	Chung-buk	1	ref		
LN metastasis	No	0.29	0.24	0.34	<0.001
	Yes	1	ref		
HG	G1	0.78	0.23	2.57	0.68
	G2	1.39	0.44	4.38	0.56
	G3	4.33	1.39	13.52	0.011
LVI	No	0.31	0.26	0.36	<0.001
	Yes	1	ref		
Ki-67	No	0.21	0.13	0.34	<0.001
	Yes	1	ref		
		HR	95% CI		p-valued
HER2 overexpression	Negative	0.26	0.16	0.43	<0.001
	Positive	1	ref		
Radiotherapy	No	1.16	0.97	1.39	0.08
	Yes	1	ref		
Chemotherapy	No	0.47	0.39	0.58	<0.001
	Yes	1	ref		
Endocrine therapy	No	3.28	2.75	3.93	<0.001
	Yes	1	ref		
IC	No	1	ref		<0.004
	Yes	7.41	1.85	29.66	

HRT, Hormonal Replacement Therapy; HR, Hazard Ratio; CI, Confidence Interval; LN, Lymph node; HG, Histologic Grade; LVI, Lymphvascular invasion; HER-2; Human Epidermal Receptor-2/neu; IC, Interval Cancer ^d^p values were calculated using the Х^2^ and t-tests.

We observed significant correlations between ICs among women with a family history of breast cancer. In concordance with this, Holm et al., (2015) reported a two-fold increase in the odds for ICs among BRCA mutation carriers, in line with our results. Other studies of BRCA mutations have reported a lowered sensitivity of mammography screening for carriers (Brekelmans et al., 2001; Komenaka et al., 2004). However, previous literature (Domingo et al., 2010; Kirsh et al., 2011; Musolino et al., 2012; Blanch et al., 2014) on family history and ICs reports conflicting results, using varying definitions of family history and low patient numbers. We observed a small effect of family history on interval cancer, but results require confirmation in larger studies. 

In particular, educational level is an important health determinant and is an important predictor of participation in certain types of cancer screening. In a meta-analysis, Damiani et al., (2012) reported a positive association between education level and adherence to breast cancer screening. Other studies (Damiani et al., 2012; Martín-López et al.,2012) observed that women with a lower level of education tended to adhere more to organized screening programs than to opportunistic ones. Educational level was a strong independent prognostic factor in other Korean breast cancer studies and Hwang et al., (2017) reported that a high education level conferred a superior prognosis compared to that of a low educational level which was consistent with this study. High educated women were frequently diagnosed as ICs, but the prognosis of them was better. Many factors could be related to this correlation at both at contextual level, such as the type of health care provider, the accessibility to care, and at the individual level such as age, location, income, private insurance coverage status, and occupational status.

The distribution of tumor characteristics between ICs and non-ICs overall was not in full agreement with other literature, with ICs being larger at diagnosis (Komenaka et al., 2004; Brekelmans et al., 2001), of higher grade (Domingo et al., 2010; Kirsh et al., 2011; Meshkat et al., 2015), and displaying more lymph node involvement (Musolino et al., 2012; Blanch et al., 2014) and more often being ER/PR-negative (Damiani et al., 2015; Musolino et al., 2018), HER2-positive (Martín-López et al., 2012; Musolino et al., 2018), or triple negative (Gilliland et al., 2000). As results, these studies have reported that IC tumors had, on average, a poorer prognosis of ICs than those of screening-detected breast cancers (Andersson et al., 1998; Collett et al., 2005; Sihto et al., 2008). While 5 years overall survival of ICs is better than non- ICs who have ever participated in breast screening in the current study, in addition to favorable features. To highlight the cause of differences in molecular characteristics of IC compared to other studies, large-scaled prospective studies or a centralized tumor bank are needed. Previous studies have reported differences in molecular characteristics between ICs and screening-detected breast cancers. Rojo et al., (2014) showed differential expression profiles both at gene and protein levels, especially mTOR signaling, which is upregulated in true interval cancers, suggesting that this pathway may mediate aggressiveness. Li et al., (2015) first reported that these two types of breast cancer may have unique underlying biology based on a 77 single-nucleotide polymorphism risk score However, a recent, well-designed study indicated molecular differences between less aggressive screening-detected breast cancer and more aggressive ICs. The two diseases are biologically distinct in terms of somatic mutations, copy number aberrations, and gene expression, but most of these differences are no longer significant after adjusting for breast cancer subtypes and mammography density (Li et al., 2017).

Province was concerned as associated factors for OS. Specific location (Kyung-nam province) need improvement in prognosis which could conceivably be attained through increased public education and awareness of interval breast cancer. In 2012, Disparities on cancer screening or cancer survival were reported, consistent with present study (Khang and Lee, 2012). Based on actual short term survival rather than actual short term DFS, the present results are likely to be due to socio-economic factors such as income and educational level rather than treatment differences for breast cancer. In order to reduce this disparity, various institutional and non-institutional devices need to be prepared and operated in Korea.

This study has several limitations. First, the study assessed several associated host factors including age, family history, hormone replacement therapy, residence, and educational level, but it did not assess their independent contributions. Second, because of short follow-up and low mortality rate, we could not perform a stratified analysis between the IC and non-IC groups. Third, the proportion of IC from registry dataset was relatively lower than that reported in other studies (Kalager et al., 2012; Bellio et al., 2017; Delacour-Billon et al., 2017; Musolino et al., 2018) or in the health insurance claim data (Lee et al., 2016). The reason is that the source of data is not from a national cancer screening dataset but a cancer registry that includes 81.1 % of all incident breast cancers (Oh et al., 2016). The registry is based on voluntarily participating breast surgeons of teaching hospitals in Korea. Thus a considerable number of study participants with missing values can be expected and selection bias is possible. This data also depend on diagnosis of ICs by physicians, and unpredictable under- diagnosis may happen. However, many studies have reported high correlations between the rates derived from chart audits and patient surveys (Montano et al., 1995; Hoffmeister et al., 2007). Nevertheless, this study has several strengths compared to the national breast cancer screening database. First, data from physician or patient interviews have more definitive categorization than those of other retrospective observational studies because these data excluded end-stage breast cancer patients who received only palliative treatment with no anti-cancer treatment or surgery. Second, the present study included more social and environmental factors to assess their relationship with ICs in Korea. Because of rapid increasing breast cancer risk factors in Korea, such as having lower child-birth (if any) at an older age and high educational level, these related factors could play role on the occurrence of ICs as limitation of screening. 

In conclusion, among women with breast cancer who have ever undergone breast screening, ICs and non-ICs showed disparate clinicopathologic features and regional or educational disparities. While the mammography-based screening has been helpful for the early detection of breast cancer, detection of ICs is a limitation with decreased examinee compliance. Nevertheless, the proportion of IC was very low in KBCR and the short-term survival was significantly better than non-ICs. 

## Funding statement

This work was supported by a grant from Research Year of Inje University in 2017. 

## Data Availability

The Korean Breast Cancer Registry restricts their dataset by internal regulation by the Korean Breast Cancer Society. This article was permitted to access the registry by the Korean Breast Cancer Society (approval no: WA 604-20170-724-01).
